# Elevated ZNF704 expression is associated with poor prognosis of uveal melanoma and promotes cancer cell growth by regulating AKT/mTOR signaling

**DOI:** 10.1186/s40364-023-00471-y

**Published:** 2023-04-10

**Authors:** Jingting Luo, Haowen Li, Jingying Xiu, Jingyao Zeng, Zhaoxun Feng, Hanqing Zhao, Yang Li, Wenbin Wei

**Affiliations:** 1 Beijing Key Laboratory of Intraocular Tumor Diagnosis and Treatment, Beijing Ophthalmology & Visual Sciences Key Lab, Medical Artificial Intelligence Research and Verification Key Laboratory of the Ministry of Industry and Information Technology, Beijing Tongren Eye Center, Beijing Tongren Hospital, Capital Medical University, Beijing, 100730 China; 2 National Genomics Data Center, Beijing Institute of Genomics, Chinese Academy of Sciences, Beijing, 100101 China; 3 Department of Ophthalmology, University of Ottawa, 501 Smyth Rd, Ottawa, ON K1H 8M2 Canada

**Keywords:** Uveal melanoma, ZNF704, SORBS3, AKT/mTOR

## Abstract

**Background:**

Uveal melanoma (UM) is the most common intraocular malignancy in adults, with a poor survival prognosis. To date, limited understanding of UM’s molecular mechanisms constitutes an obstacle to developing effective therapy. In this study, we examined key regulators mediating UM progression and their clinical relevance.

**Methods:**

Transcriptomics of UM patients and cells were analyzed via RNA sequencing and bioinformatic analysis. Zinc finger protein 704 (ZNF704) was identified as prognosis-related biomarker for UM based on clinical characteristics and RNA-seq data from The Cancer Genome Atlas (TCGA). Gene expression was knocked down by specific shRNAs/siRNAs and overexpressed by transfection with plasmids inserted with investigated gene cDNA. Cell proliferation, viability and invasion abilities were determined by CCK8, colony formation and transwell assays, respectively. For cell cycle and apoptosis, cells were PI or PI/Annexin V-APC stained and analyzed by flow cytometry. Standard immunoblotting and quantitative RT-PCR were employed to assess the mRNA and protein abundance. To determine tumor growth in vivo, 4-week-old BALB/c-nu immune-deficient nude mice were inoculated with tumor cells.

**Results:**

Analysis of differential expressed genes (DEGs) and survival analysis identified ZNF704 as a novel biomarker of UM. Prognostic analysis indicated ZNF704 as an independent predictor of UM overall survival. Expression of ZNF704 is elevated in UM tissues relative to adjacent normal choroid tissues. Knockdown of ZNF704 suppressed the growth and migration of UM cells and vice versa. In addition, expression of ZNF704 arrest UM cells at G0/G1 phase and inhibit cell apoptosis. RNA sequencing analysis indicated that SORBS3 were dysregulated after ZNF704 downregulation. Gene Set Enrichment Analysis (GSEA) revealed that upon ZNF704 knowndown, genes related with PI3K/AKT/mTOR, EMT and metastasis are enriched. Mechanistically, ZNF704 activates AKT/mTOR/glycolysis signaling pathway in UM cells. Moreover, expression of SORBS3 is downregulated by ZNF704 and knockdown of SORBS3 restored tumor cell viability in ZNF704 silenced cells.

**Conclusions:**

ZNF704 predicts poor prognosis of UM and exhibit pro-oncogenic effect in UM progression in vivo and in vitro, mediated through AKT/mTOR signaling pathway and suppression of SORBS3 expression.

**Supplementary Information:**

The online version contains supplementary material available at 10.1186/s40364-023-00471-y.

## Background

Uveal melanoma (UM) is an ocular subtype of melanoma arose from uveal melanocytes with an worldwide incidence of 5.1 per million [1, 2]. Although excellent local control can be achieved through radiation or enucleation, UM mortality is high due to lethal metastasis and the lack of effective systemic treatment [3]. An active area of research is to identify molecular drivers of UM based on genomics and epigenomics analysis. Mutations of G protein subunit alpha q (GNAQ) or G protein subunit alpha 11 (GNA11) are well-established primary disease drivers for UM [4, 5]. Epigenetic changes such as DNA methylation and histone modifications also have been shown to contribute to UM tumorigenesis [6]. During the past decades, transcriptomics and proteomics are widely employed to identify novel oncogenes or tumor suppressors in cancer progression. The Cancer Genome Atlas (TCGA) which is primarily based on RNA sequencing of different primary cancer types has greatly contributed to progress on cancer research.

Zinc-finger protein family, the largest transcription factor family, exhibits a multitude of functions in human disease through transcriptionally activates or suppresses expression of downstream targets [7]. Zinc finger protein 704 (ZNF704) is part of the zinc-finger protein family and is mapped on 8q21. Prior studies suggested that up-regulation of ZNF704 gene was found to promote carcinogenesis in breast cancer [8]. In addition, ZNF704 interacted with SIN3A complex to transcriptionally inhibit the expression of PER2, which is an important circadian rhythm regulator. Nevertheless, the role of ZNF704 in most cancers including UM is largely unknown.

The present study aimed to identify reliable UM biomarker that associates with prognosis. We investigated the prognostic significance of ZNF704 using data from TCGA database and performed preliminary functional experiments to validate its roles in vivo and in vitro and transcriptomic analysis to reveal its molecular mechanism. Our study provides insight to a novel prognostic marker, ZNF704, that may be explored as a therapeutic target in future experiments.

### Methods

#### UM patients

A total of 5 paired UM tissues and matched adjacent normal choroidal tissues for transcriptomic analysis were collected from Tongren Hospital, Capital Medical University. Experiment protocols were approved by the Clinical Research Ethics Committee of Tongren Hospital, Capital Medical University in accordance with the declaration of Helsinki. The samples were collected before any interventions and a written informed consent was given by each patient. UM and normal tissues were subjected to transcriptomics analysis, following the manufacturer's instructions.

### The Cancer Genome Atlas (TCGA)

Correlation between ZNF704 expression and UM patients’ survival was analyzed from The Cancer Genome Atlas website (http://cancergenome.nih.gov).

### Identification of prosnosis-related genes

Based on the RNA-seq data of 80 samples from the TCGA-UM dataset, “limma” package v3.48.3 in R was used to assess differential gene expression in the metastasis and non-metastasis groups. The criteria for selecting differentially expressed genes (DEGs) included log2 fold change|> 2 and a false discovery rate (FDR)-adjusted *p* < 0.05. Results from these analyses were plotted as volcano plots using the “ggplot2” package v3.3.5 in R.

### Survival analysis and assessment of ZNF704 for prognostic prediction

The prognostic impact of ZNF704 expression in several types of cancer was investigated by pan-cancer analysis. RNA-seq data and clinical information were downloaded from the TCGA database (https:// portal.gdc.cancer.gov/). Patients in the TCGA cohort were categorized into those with high and low levels of expression of ZNF704 based on the median level of ZNF704. OS in the TCGA cohort was analyzed by the Kaplan–Meier method (significance level: *p* < 0.05), using the “survival” package v3.2–13 in R. Bulk RNA-seq from TCGA database were used for the univariate and multivariate cox hazard analysis of association between the expression of ZNF704 and UM patients’ prognosis, with the results displayed as forest plots using the “forestplot” package v2.0.1 in R. The predictive power of the model was evaluated using receiver operating characteristic (ROC) and Cox regression (significance level: *p* < 0.05) analyses, as calculated using the R packages “survival” v3.2–13, “survminer” v0.4.9 and “timeROC” v0.4. In addition, because cytogenetic studies have found loss of chromosome 3 (monosomy 3) to be associated with UM metastasis and the status of chromosomes 3 has been determined in every patient in the TCGA-UM project. Mann–Whitney test was used to analyze the association between ZNF704 expression and chromosome 3 status (significance level: *p* < 0.05). Univariate Cox regression (significance level: *p* < 0.05) analysis was used to assess the relationship between OS and ZNF704 expression, with the results displayed as forest plots using the “forestplot” package v2.0.1 in R.

### Immunohistochemistry (IHC)

Tissues were fixed in 4% paraformaldehyde, washed three times with PBS, transferred to 70% ethanol, then embedded in paraffin and sectioned according to standard procedures. Sections were dewaxed with a graded ethanol series. After antigen retrieval, the tissues were stained using the Streptavidin Peroxidase IHC assay kit (ZSGB-Bio, Beijing, China), using 3-amino-9-ethylcarbazole (AEC) and 3,30-diaminobenzidine tetrahydrochloride (DAB) as chromogens, hematoxylin was used for the re-staining of nucleus.. The primary antibodies were anti-ZNF704 (1:200, Thermo Fisher Scientific, Massachusetts, USA), anti-SORBS3 (1:200, Thermo Fisher Scientific, Massachusetts, USA), anti-KSR2 (1:100, Abnova, Taiwan, China), anti-14–3-3ζ (1:800, CST, Massachusetts, USA), anti-ERK1/2 (1:400, CST, Massachusetts, USA, anti- Cleaved-Caspase 3 (1:100, CST, Massachusetts, USA) and anti-KI67 (1:100, Abcam, Cambridge, UK). The average red (AEC) /gray (DAB) value of each image was used to quantify the expression level using Image-Pro Plus 6.0 software.

### Cell culture

UM cells, including OCM1A and C918, were from American Type Culture Collection (Manassas, VA, USA). Cells were maintained in RPMI 1640 medium, which was supplied with 10% heat-inactivated fetal bovine serum (FBS), HEPES buffer, L-glutamine, MEM essential vitamine mixture, MEM non-essential amino acid, and 1% antibiotics under 37 oC with 5% CO2, as described previously [9].

### ZNF704 and SORBS3 knockdown

To knock down ZNF704, we purchased shRNAs against negative control and ZNF704 from Beijing Syngentech Co., Ltd, China. shRNAs were transfected into UM cells. 48 h later, knockdown efficiency was detected by quantitative real-time PCR (qRT-PCR) and immunoblotting. Then cell proliferation, colony growth and migration were assessed in these cells. SORBS3 was knocked down by siRNAs. The sequence of shRNA and siRNA was as follow: shZNF704-1: 5’-GCAATCTCCTCCGGTCACTTT-3’; shZNF704-2: 5’-GGATGGAGAACCGAGACATGT-3’; shCtrl: 5’-TTCTCCGAACGTGTCACGT-3’; siSORBS3-1: 5’-GCATCTTCCCTGCTAATTA-3’; siSORBS3-2: 5’-CCCAGAAATTCGGAACGTT-3’; siCtrl: 5’-TTCTCCGAACGTGTCACGT-3’.

### ZNF704 ectopic expression

ZNF704 over-expressing lentivirus were purchased from Beijing Syngentech Co., Ltd, China. In brief, coding sequence of ZNF704 was synthesized and inserted into lentivirus over-expressing vector. Lentivirus were packaged in 293FT cells and were concentrated by ultracentrifugation. After concentration, lentivirus was used to infect UM cells to over-express ZNF704. 48 h later, knockdown efficiency was detected by quantitative real-time PCR (qRT-PCR) and immunoblotting. Cell proliferation, colony growth and migration were assessed in these cells.

### RNA isolation and qRT-PCR

Total RNA was isolated from UM cells using the Trizol reagent, according to manufacturer's protocols. RNA quantity and quality were measured by NanoDrop 1000. RNA was reversely transcribed to cDNA using PrimeScript™RT Master Mix (Takara, RR036Q), following the manufacturer's protocols. ZNF704 transcript abundance and other genes was detected by using Promega GoTaq® qPCR Master Mix (Promega, A6001) on the qPCR machine. Primer sequence was as forward: ZNF704 forward, 5’-GATCAAGCTCAACACAGACTCA-3’, and reverse, 5’-TCTGGGATGGGGAAAGTAGGA-3’; β-actin forward, 5’-CATGTACGTTGCTATCCAGGC-3’, and reverse, 5’-CTCCTTAATGTCACGCACGAT-3’.

### Immunoblotting

Total proteins were isolated from UM cells using RIPA buffer, according to the manufacturer's protocols. Concentration of total proteins was detected by BCA kit. 50 ug of the proteins were separated on SDS- polyacrylamide gel electrophoresis and immunoblotting on PVDF membranes. After blocking with 5% non-fat milk, incubating with primary and secondary antibodies, protein abundance was detected on chemiluminescence system. ZNF704 primary antibody was obtained from Invitrogen. β-actin primary antibody was obtained from Santa Cruz. All the secondary antibodies were from Proteintech.

### CCK8

After knocking down or over-expressing ZNF704, UM cells were plated in 96-well plates in triplicate at the concentration of 2000 cells per well. After adding CCK into each well and incubating for 2–4 h at 37 ℃, viable cells were measuring OD450.

### Colony growth

After knocking down or over-expressing ZNF704, UM cells were plated in 6-well plates in triplicate at the concentration of 500 or 1000 cells per well. 7–10 days later, each well was washed with PBS for three times and the colonies were fixed by methanol for 30 min. Crystal violet was then used to stain the colonies. The pictures of colonies were collected by the camera.

### Cell cycle

PI staining was used to analyze cell cycle. UM cells was washed by PBS and fixed in 70% iced alcohol overnight. After stained by PI, cell cycle was measured on a flow cytometry system.

### Apoptosis

PI/Annexin V staining was used to detect apoptosis. UM cells were washed by PBS and immediately stained by PI and Annexin V. Then apoptosis was analyzed on the flow cytometry system.

### Migration

After knocking down or over-expressing ZNF704, UM cells in FBS-free culture medium were plated on the upper surface of transwell chambers. 500 μl culture medium containing 10% FBS was added into lower well. 24 h later, UM cells on the upper surface were removed and UM cells on lower surface was fixed by methanol and stained by crystal violet. Migrated cells were photographed under the microscope.

### Chromatin Immunoprecipitation (ChIP)

ChIP was performed using the ChiP Assay Kit according to the manufacturer’s instructions (N259-YH01, Novoprotein). The nucleus was isolated, and the chromatin was sonicated and immunoprecipitated with anti-FLAG antibodies (ChIP +) or normal rabbit IgG (ChIP–, negative control). The reaction system was as follows: 1 µL of ChIP product, 1.0 µL of each forward or reverse primer, and 10 µL of qPCR Master Mix (Sangon Biotech, Shanghai, China), with a total volume of 20 µL. The thermal profiles were 40 cycles of 95 °C for 15 s, 60 °C for 30 s, and 72 °C for 30 s. The primers used for ChIP-PCR are shown in Supplementary Table 1.

### Animal experiment

BALB/c-nu mice (4-week-old) were randomly divided into two groups. Indicated cells were injected subcutaneously into each mouse. Every 3 days, Xenografts were examined and size measured. 34 days later, animals were euthanized, and tumors were excised and weighed. Animal experiment included in this study agreed with the Ethics Committee of Beijing Tongren Hospital (TRECKY2018–056).

### Statistical analysis

Statistical significance was analyzed by GraphPad prism software. The results were shown as mean ± SEM (n = 3). Student’s t test was used to determine the difference between two groups. *P* < 0.05 was considered statically significant.

## Results

### Prognosis-Related Hub Genes in UM

**Fig. 1:**
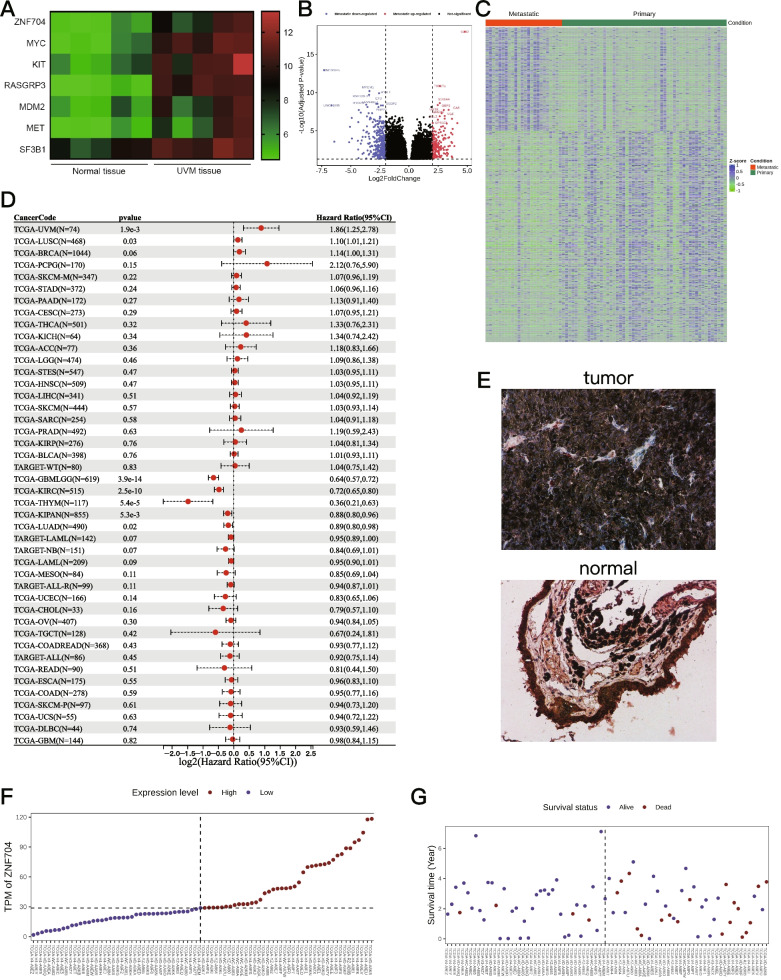
Identification of ZNF704 as a biomarker in UM. **A** Transcriptomics analysis of genes dys-regulated in UM tissues as compared to normal tissues. **B** The volvano plot showing analysis of DEGs based on metastasis status in TCGA cohort (FDR < 0.05, | log2(fold change)|> 1). **C** Heatmap of gene expression in two groups divided according to metastasis status. **D** Pan-cancer analysis indicating that multiple cancer types were associated with ZNF704 expression. **E** IHC staining of ZNF704 in tumor (top) and adjacent normal (bottom) tissues. **F** Distribution of ZNF704 expression levels in the TCGA corhort. The dotted line represented the median ZNF704 expression and divided the patients into low-expression and high-expression groups. **G** Percentage of deceased patients in the TCGA cohort as a function of ZNF704 expression level

5 UM tissues and 5 adjacent normal uveal tissues were collected and subjected to transcription analysis of dysregulated genes. Dysregulated genes were defined using the criteria: fold change > 1.5 and *p* < 0.05 (Supplementary Table 2). Relative to normal tissue, we identified 6 down-regulated and 53 up-regulated genes in UM tissues (Fig. 1A). Among these genes, ZNF704, MYC, KIT, RASGRP3, MDM2, MET and SF3B1 were significantly over-expressed in UM tissues (Fig. 1A). Considering metastasis is one of the main factors affecting the prognosis of UM, we assigned 80 TCGA-UM samples into two groups according to their metastasis status to analyze DEGs related to tumor progression (Fig. 1B and C). Among 7 over-expressed genes, only ZNF704 were up-regulated in metastasis group compared to non-metastasis group. In addition, pan-cancer analysis revealed that ZNF704 was associated with tumor progression in several cancer types (Fig. 1D). Since ZNF704 is located on chromosome 8, which is known to have prognostic significance for UM, we focused our study on the mechanism of ZNF704. Immunohistochemistry (IHC) further validated that ZNF704 was highly expressed in tumors and low in adjacent tissues (Fig. 1E). The percentages of deceased patients were greater in the high than in the low ZNF704 expression group (Fig. 1F and G).

### Association between ZNF704 expression and prognosis in patients with UM

**Fig. 2:**
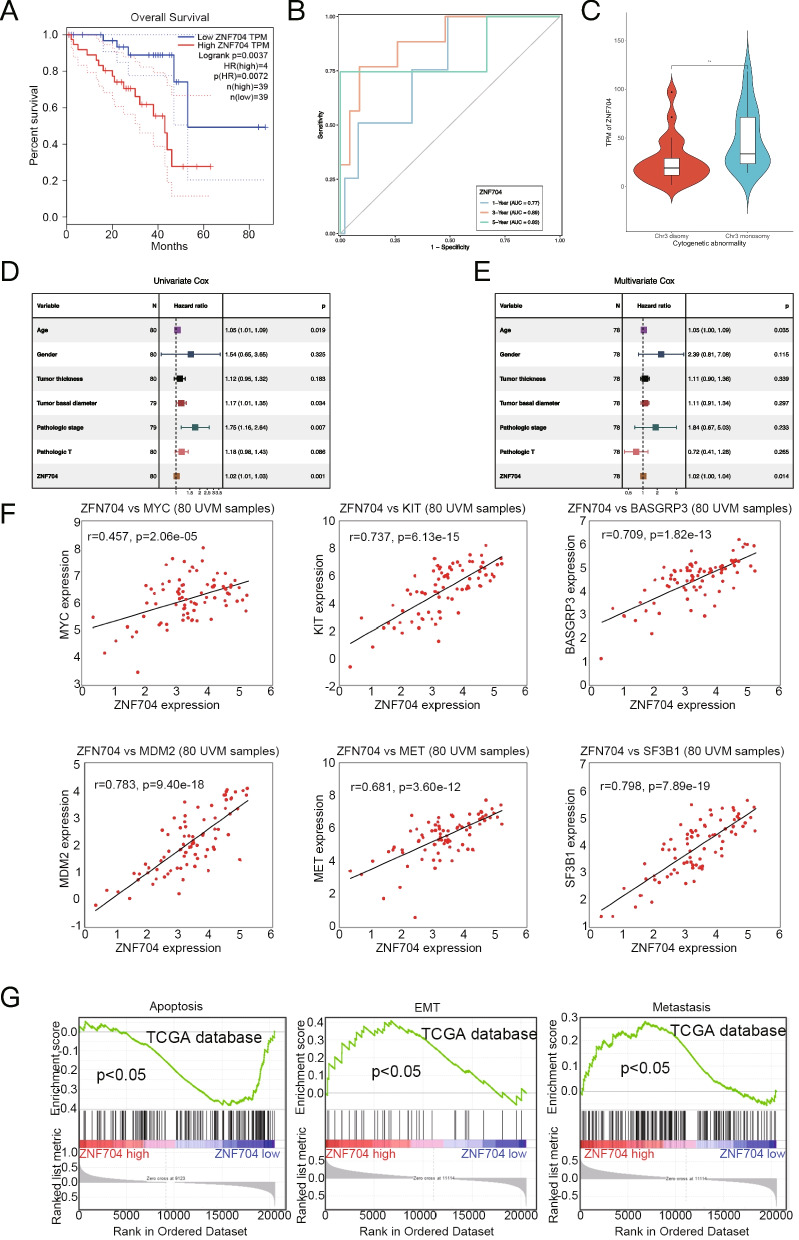
Up-regulation of ZNF704 is correlated with UM progression. **A** Overall survival of UM patients who were divided into ZNF704 high (*n* = 40) and low expression (*n* = 40) group. **B** Time-dependent ROC analysis for prediction of OS in the TCGA cohort. **C** Correlations between ZNF704 expression and chromosome 3q copy number, the density curve on the x and y-axes represents the distribution trend of themselves. The blue line indicates the best fitted linear models and the shaded area indicates the 95% confidence interval. **D** and **E** Univariate and multivariate Cox analyses of ZNF704 expression and other clinicopathological parameters in the TCGA cohort. **F** The correlation between ZNF704 and MYC, KIT, RASGRP3, MDM2, MET and SF3B1 was analyzed in UM tissues from TCGA database. Correlation value and statistical significance were shown in the figure. **G** GSEA showed that ZNF704 high expression was correlated with inactivated apoptosis, and activated EMT and metastasis gene sets in UM samples

We analyzed the correlation between ZNF704 and UM patients’ prognosis from TCGA database. We found UM patients with high ZNF704 expression exhibited poorer survival than those with ZNF704 low expression (Fig. 2A). The worse prognosis remain true after adjusting for age, gender, or tumor stages, suggesting that ZNF704’s pro-oncogenic role was irrespective of other factors (Supplementary Fig. 1). Time-dependent ROC curves were constructed to assess the prognostic value of ZNF704 in predicting 1-, 3-, and 5-year OS based on TCGA data. The AUC values for predicting 1-, 3-, and 5-year OS of patients with UM were 0.77, 0.89, 0.83, respectively, as outlined in Fig. 2B. Since the loss of chromosome 3 (monosomy 3) was associated with UM metastasis, we further verify the correlation between ZNF704 expression and chromosome 3 status (Fig. 2C). Univariate Cox analysis (Fig. 2D) and multivariate Cox analysis (Fig. 2E) further showed that ZNF704 was independently prognostic of survival in patients with UM. ZNF704 expression was also positively correlated with the expression of MYC, KIT, RASGRP3, MDM2, MET and SF3B1 in UM samples based on TCGA database (Fig. 2F). Gene Set Enrichment Analysis (GSEA) found that apoptosis activation genes were enriched in low ZNF704 expression UM samples, while genes related to EMT and metastasis activation were enriched in high ZNF704 expression UM samples (Fig. 2G). Taken together, ZNF704 over-expression is likely a biomarker for UM progression and metastasis.

### ZNF704 knockdown inhibits UM cell growth

**Fig. 3:**
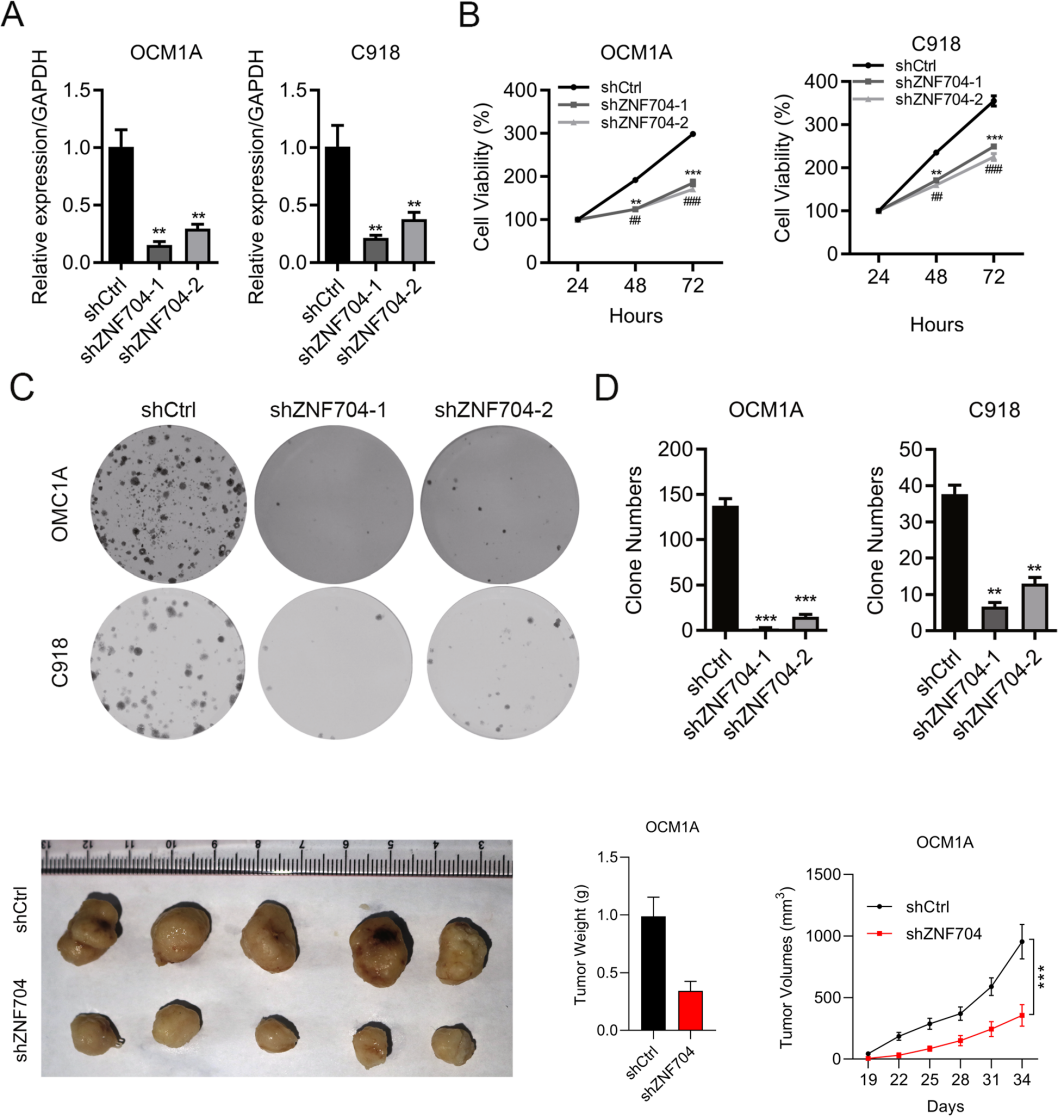
ZNF704 knockdown suppresses UM cell growth. **A** qRT-PCR analysis of ZNF704 in shCtrl, shZNF704-1 and shZNF704-2 OCM1A and C918 cells. **p < 0.01. **B** CCK8 assay was used to detect cell growth in shCtrl, shZNF704-1 and shZNF704-2 OCM1A and C918 cells. ** or ##*p* < 0.01. *** or ###*p* < 0.001. **C** and **D** Colony formation was assessed in shCtrl, shZNF704-1 and shZNF704-2 C918 and OCM1A cells. **p < 0.01. ***p < 0.001. **E**–**G** ZNF704 knockdown inhibits xenograft growth in vivo

We further investigated the association between ZNF704 and UM growth. shRNAs was used to knock down ZNF704. qRT-PCR and immunoblotting results demonstrated that ZNF704 was down-regulated in shZNF704-1 and shZNF704-2 UM cells (Fig. 3A). CCK8 results showed that ZNF704 knockdown suppressed the growth of OCM1A and C918 cells (Fig. 3B). Furthermore, the numbers of colonies were decreased in ZNF704 silenced C918 and OCM1A cells (Fig. 3C and D). In addition, Xenograft growth in vivo was significantly inhibited in ZNF704 knockdown group compared to the control group, indicating that ZNF704 is involved in UM tumorigenesis (Fig. 3E-G).

To further validate the role of ZNF704 in UM, we over-expressed ZNF704 by using lentivirus. qRT-PCR results demonstrated that ZNF704 was efficiently over-expressed in OCM1A and C918 cells (Supplementary Fig. 2A). Over-expression of ZNF704 promoted the proliferation of UM cells (Supplementary Fig. 2B). The numbers of colonies increased in ZNF704 over-expressed C918 and OCM1A cells (Supplementary Fig. 2C and 2D). In addition, ZNF704 ectopic expression reduced UM cell apoptosis (Supplementary Fig. 2E and F). These results suggest that ZNF704 expression promotes UM cell growth.

### ZNF704 knockdown inhibits UM cell growth by inducing cell cycle arrest and apoptosis

**Fig. 4:**
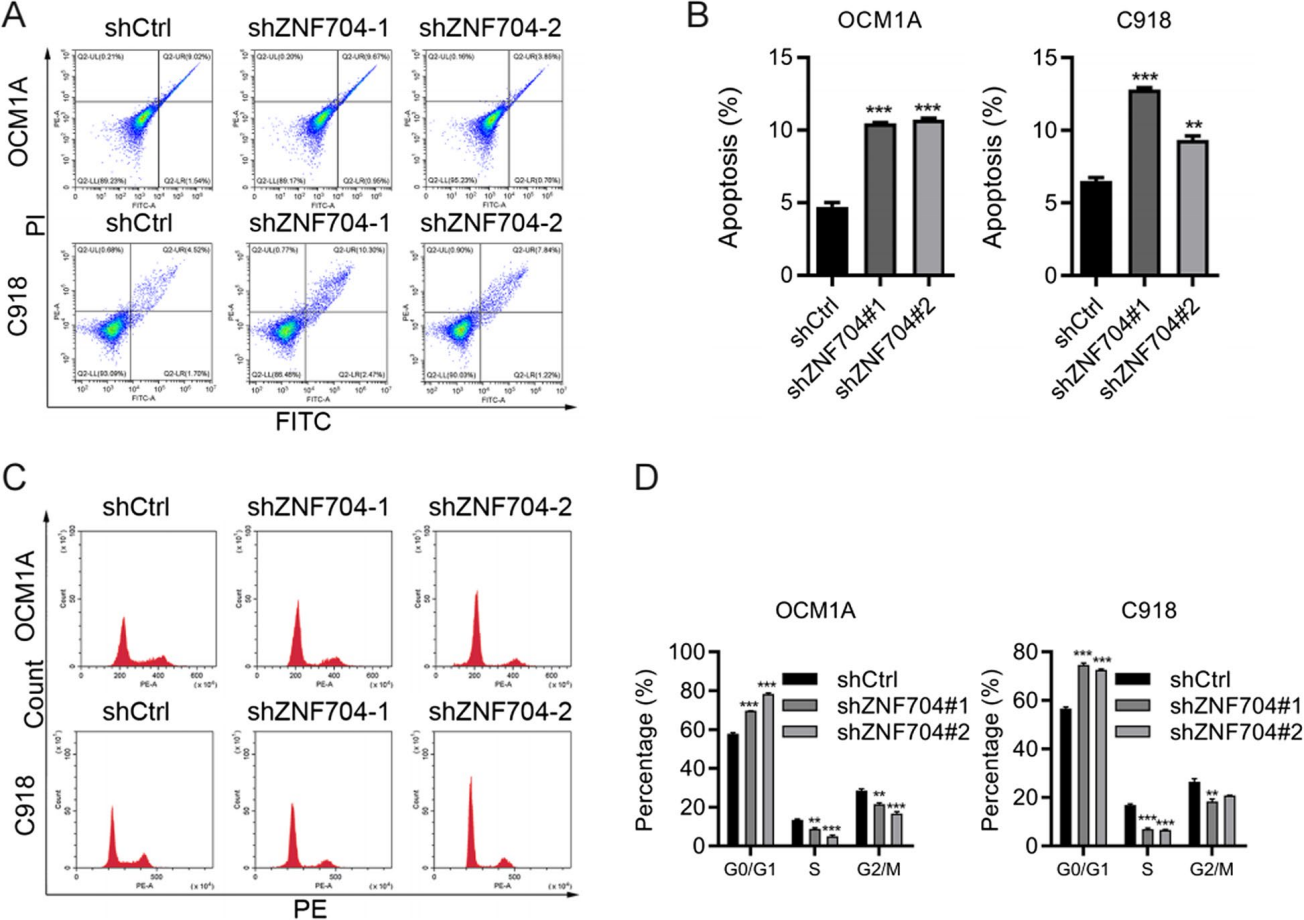
ZNF704 regulates apoptosis and cell cycle in UM cells. **A** and **B** Apoptosis was analyzed by PI/Annexin V staining in shCtrl, shZNF704-1 and shZNF704-2 C918 (A) and OCM1A (B) cells. ***p < 0.001. **C** and **D** Cell cycle was analyzed by PI staining in shCtrl, shZNF704-1 and shZNF704-2 C918 (C) and OCM1A (D) cells. *p < 0.05. **p < 0.01

We then analyzed the effect of ZNF704 on apoptosis and cell cycle distribution in UM cells. PI/Annexin V staining was used to analyze apoptosis after ZNF704 knockdown in UM cells. As shown in Fig. 4A and B, apoptosis was enhanced after ZNF704 knockdown in both C918 and OCM1A cells. ZNF704 knockdown led to decreased G0/G1 phase, increased S and G2/M phase in C918 cells. For OCM1A cells, ZNF704 down-regulation resulted in increased G0/G1 phase, decreased S and G2/M phase (Fig. 4C and D). These results indicated that ZNF704 silencing induced G2/M cell cycle arrest in C918 cells and G0/G1 cell cycle arrest in OCM1A cells. Overall, ZNF704 knockdown promotes apoptosis and cell cycle arrest in UM cells.

### ZNF704 promotes UM cell migration and epithelial-to-mesenchymal transition

**Fig. 5:**
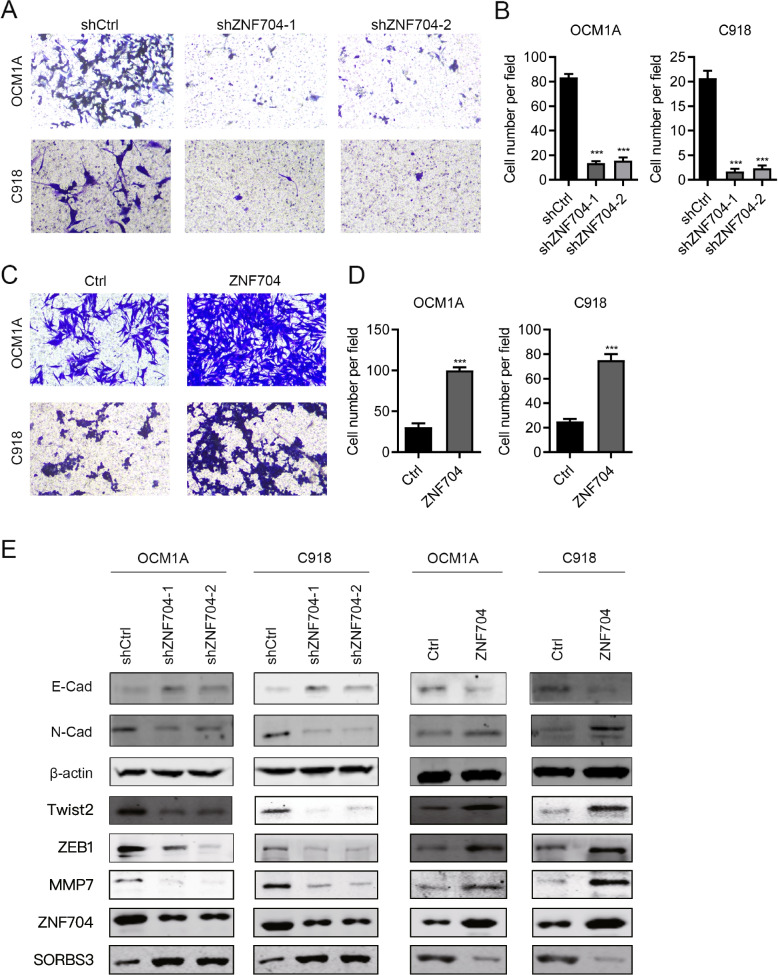
ZNF704 promotes UM cell migration and EMT. **A** and **C** Transwell was used to detect migration in shCtrl, shZNF704-1 and shZNF704-2 C918 and OCM1A cells. ***p < 0.001. **B** and **D** Transwell was used to detect migration in Ctrl and ZNF704 over-expressed C918 and OCM1A cells. ***p < 0.001. **E** and **F** ZNF704 knockdown and over-expressed C918 and OCM1A cells were subjected to immunoblotting with indicated antibodies

Over half of UM patients has liver metastasis. We aimed to study the role of ZNF704 in UM metastasis other than in situ cell growth. Transwell assays were performed in C918 and OCM1A cells after ZNF704 knockdown and over-expression. We found that ZNF704 knockdown suppressed the migration capacity of C918 and OCM1A cells (Fig. 5A and B). Conversely, ZNF704 ectopic expression led to enhanced migration in C918 and OCM1A cells (Fig. 5C and D). Epithelial-to-mesenchymal transition (EMT) is an important event in cancer progression and metastasis. We evaluated whether ZNF704 regulated EMT in UM. Immunoblotting results showed that E-cadherin was up-regulated in ZNF704 knockdown and was down-regulated in ZNF704 over-expressed UM cells and N-cadherin was positively regulated by ZNF704 (Fig. 5E and F). Thus, we demonstrated that ZNF704 contributes to UM cell migration and EMT process in vitro.

### ZNF704 regulates AKT/mTOR signaling pathway

**Fig. 6:**
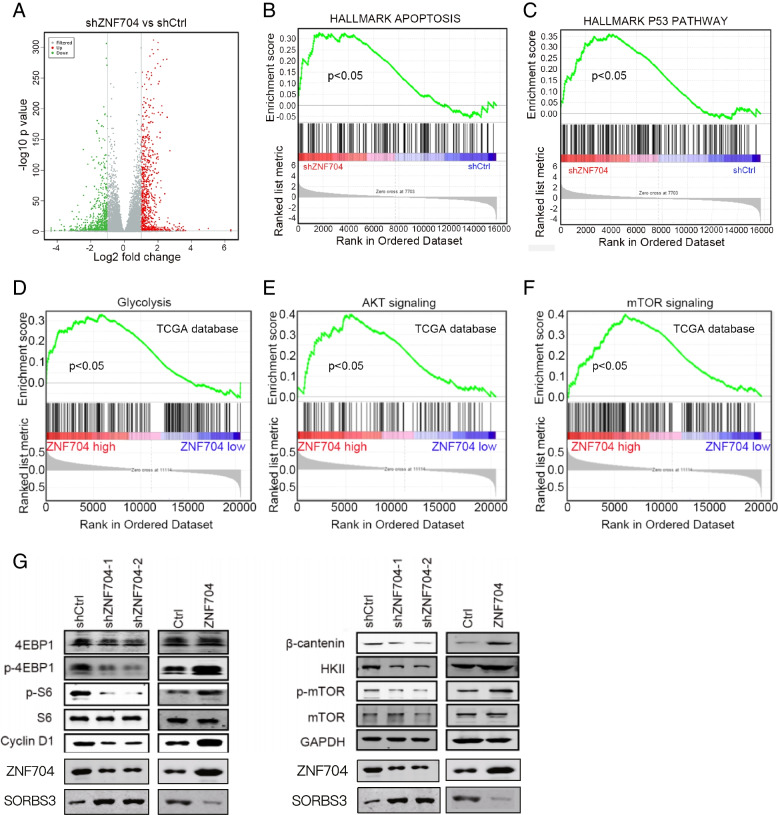
Downstream targets of ZNF704 in UM. **A** Total RNA was isolated from shCtrl and shZNF704 cells and subjected to RNA sequencing analysis. Heatmap of dys-regulated genes. **B** and **C** GSEA showed that apoptosis and P53 signaling pathway was correlated with ZNF704 expression. **D**-**F** GESA showed that AKT, mTOR and glycolysis were activated in UM samples with ZNF704 high expression. **G** Immunoblotting analysis of AKT/mTOR/glycolysis in ZNF704 knockdown and over-expressed UM cells

To explore the molecular mechanism of ZNF704 in UM, we subjected shCtrl and shZNF704 cells to RNA sequencing. As shown in Fig. 6A, 1983 genes were up-regulated and 1618 genes were down-regulated after ZNF704 knockdown. GSEA suggested that apoptosis and P53 pathway was activated in shZNF704 UM cells (Fig. 6B and C). We also analyzed signaling pathway regulated by ZNF704 based on TCGA data. GSEA showed that activation of AKT, mTOR and glycolysis was enriched in UM tissues with ZNF704 high expression (Fig. 6D-F). We then evaluated whether ZNF704 regulated AKT/mTOR in UM cells. Immunoblotting revealed phosphorylation of mTOR, 4EBP1 and S6, and expression of HKII and cyclin D1 were reduced in ZNF704 knockdown and increased in ZNF704 over-expressed cells (Fig. 6G). These findings indicate that ZNF704 activates PI3K/AKT/mTOR signaling pathway in UM.

### ZNF704 down-regulation of SORBS3 promotes UM cell growth and migration

**Fig. 7:**
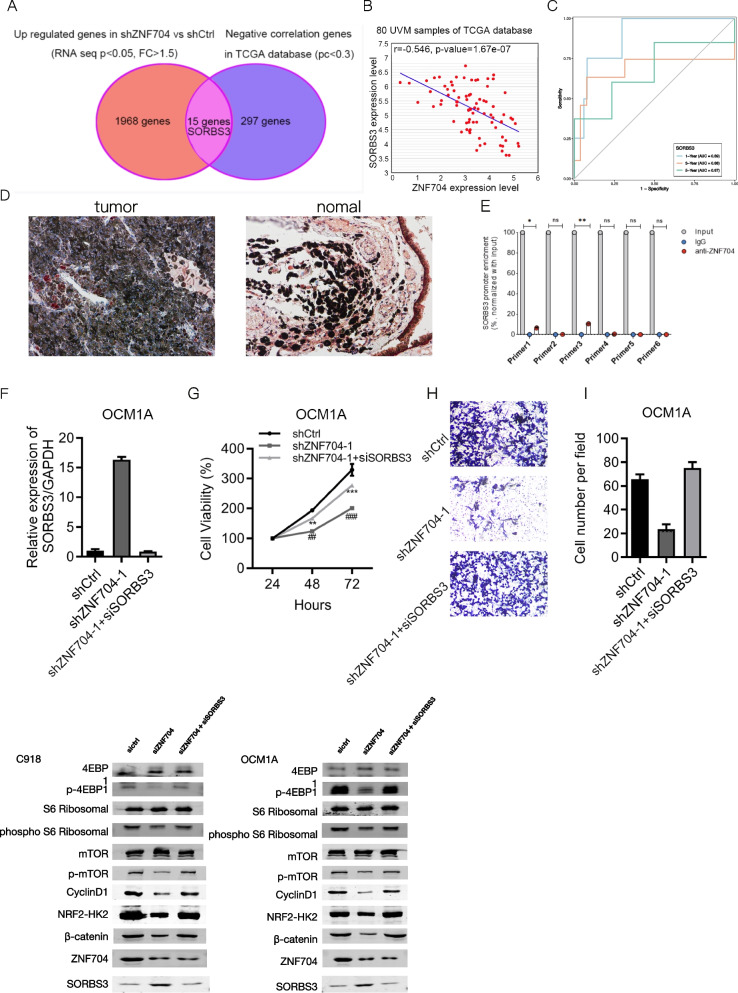
ZNF704 down-regulation of SORBS3 promotes UM cell growth and migration. **A** Overlap genes which were up-regulated in ZNF704 knockdown cells and were negatively correlated with ZNF704 in UM patients. **B** SORBS3 was negatively correlated with ZNF704 in human UM samples based on TCGA database. *r* = 0.546. *p* < 0.01. **C** Time-dependent ROC analysis for prediction of OS in the TCGA cohort. **D** IHC staining of SORBS3 in tumor (left) and adjacent normal (right) tissues. **E** Verification of the direct regulatory relationship between ZNF704 and SORBS3 using ChiP-PCR. **F** Cell migration was detected by transwell. **G** Cell proliferation was detected by CCK8 in cells as shown in C. ** or ##*p* < 0.01. *** or ###*p* < 0.001. (**H** and **I**) qRT-PCR analysis of SORBS3 in siCtrl, siZNF704-1 and siZNF704-1 + siSORBS3 OCM1A cells. **J** Western blot assay of ZNF704 and SORBS3 in siCtrl, siZNF704 and siZNF704 + siSORBS3 in C918 and OCM1A cells. **p < 0.01, ***p < 0.001

ZNF704 is a transcription factor that regulates the expression of downstream targets. Based on RNA sequencing, we showed that a variety of genes were down-regulated and up-regulated after ZNF704 knockdown. We analyzed genes negatively correlated with ZNF704 from TCGA database in human UM samples. There were 15 genes up-regulated in ZNF704 silenced UM cells and were negatively correlated with ZNF704 in human UM samples, including SORBS3 (Fig. 7A and B). The AUC values of time-dependent ROC curves for predicting 1-, 3-, and 5-year OS of patients with UM were 0.89, 0.68, 0.67, respectively, indicating that SORBS3 could effectively predict the prognosis of UM patients (Fig. 7C). We subsequently validated the expression of SORBS3 in tumor and adjacent choroid tissues (Fig. 7D). To investigate the function of SORBS3 in UM, we silenced SORBS3 in UM cells with down-regulated ZNF704. We showed that when ZNF704 knockdown reduced UM cell proliferation and migration, subsequent SORBS3 knockdown reverses the phenotypes (Fig. 7E-H). As such, we demonstrated ZNF704 mediates UM cell growth and migration through down-regulation of SORBS3.

## Discussion

UM is the most common primary intraocular malignancy in adults [3]. Most UM involves the choroid, while fewer cases arises from ciliary body and iris [10]. Genetic studies have shown that GNAQ or GNA11 mutations are the primary driver for UM tumorigenesis. To date, mortality from UM remains high due to lack of effective treatment for metastatic disease. The search for novel drug targets is the new frontier to improve prognosis of this lethal disease.

Transcription factors regulate the expression of various downstream targets and plays an important role in cancer progression. Zinc-finger proteins comprise of the largest population of transcriptional factors. High ZNF704 expression is known to predict poor prognosis in lung cancer patients [11] and ZNF704 amplification is observed in breast cancer [8]; however, little is known about its role in other cancers. In this study, we identified 7 up-regulated genes in tumor tissues compared to adjacent normal choroid tissue by RNA-seq and metastasis-related genes in TCGA dataset. Only ZNF704 overlapped in both dysregulated genes. As such, we focused our study on the effect and mechanism of ZNF704 in UM. Macroscopically, we observed patients with high ZNF704 expression had shorter OS than those with low ZNF704 expression in TCGA; this association was shown to be independent after adjustment for other factors in multivariate analysis. To explore the underlying mechanism of ZNF704’s effect on UM progression, we performed functional experiments. We demonstrated that ZNF704 expression promoted UM cell growth and migration and ZNF704 silencing induced cell cycle arrest and apoptosis. Furthermore, ZNF704 induced up-regulation and down-regulation of various genes. We further showed that ZNF704 contributes to UM cell migration and EMT process in vivo, and can up-regulated AKT/mTOR signaling pathway and down-regulated SORBS3 to promote UM cell growth and migration. Taken together, our study supports ZNF704 as an novel oncogene in UM.

EMT is a crucial step of cell transformation in many tumor types including UM [9, 12–17]. The EMT program is regulated by several zinc-finger transcription factors, including Snail [18], Slug [19, 20], zinc finger E-box binding homeobox 1 (ZEB1) [21], and SIP1/ZEB2 [22], as well as by helix–loop–helix transcriptional regulators, such as Twist [23]. Previous studies revealed that the downregulation of ZEB1 reduces the invasive properties of UM cells. The elevated mRNA levels of ZEB1 are associated with more aggressive clinical phenotype in UM samples [16]. Other zinc-finger transcription factors such as ZNF667‐AS1 were poorly expressed in UM patients with metastasis and might play an inhibitory role in the development of UM by regulating cellular aggressiveness [24]. Our study deduced ZNF704 function in promoting EMT program in UM.

PI3K/AKT/mTOR signaling pathway is one of the most frequent activated signaling pathways in cancers and promotes tumorigenesis [25, 26]. Activation of this signaling pathway is mainly caused by loss-of-function of PTEN, TSC1 and TSC2 [27–29]. Here, we showed that silencing of ZNF704 inactivated AKT/mTOR activity in UM cells, while ZNF704 over-expression activated AKT/mTOR. This suggests that ZNF704 enhances AKT/mTOR activity. Furthermore, ZNF704 is positively correlated with PI3K/AKT/mTOR signaling pathway in UM samples based on TCGA database analysis. These findings suggests that the mechanism by which ZNF704 promotes UM growth and metastasis is through activation of AKT/mTOR signaling pathway. While there’s lack of selective and potent inhibitors that target ZNF704, therapeutic targeting of PI3K/mTOR might offer a new strategy for halting progression of UM [30].

Finally, recent investigations have shown RBP sorbin and SH3 domain-containing (SORBS) proteins, including SORBS1, SORBS2 and SORBS3, exhibit tumor suppressive function in cancer growth. SORBS1 suppresses metastasis and improves chemotherapy sensitivity in cancers [31]. The most well studied SORBS protein is SORBS2, which functions as a tumor suppressor in cervical, ovarian cancer and hepatocellular carcinoma (HCC). SORBS3 has been shown to suppress HCC development by inhibiting interleukin-6 signaling [32]. In this study, we identified that ZNF704 negatively regulated the expression of SORBS3 based on RNA sequencing, qRT-PCR and immunoblotting results. Silencing SORBS3 significantly reduced the levels of p-PI3K/PI3K, p-4EBP1/4EBP1, and p-S6 ribosome/S6 ribosome. In addition, the results of Chip-PCR verified that SORBS3 is a target gene regulated by ZNF704. Thus, we hypothesize that ZNF704 could modulate the PI3K/AKT/mTOR signaling pathway via regulating the transcription of SORBS3. Loss-of-function experiments suggested that SORBS3 silencing enhanced the progression of UM cells in vitro. These findings indicate that ZNF704 promotes UM growth and metastasis by down-regulating SORBS3.

## Conclusions

In the present study, we identified ZNF704 as a prognosis-related biomarker using transcriptomic analysis and verified poor prognosis using survival data from the TCGA database. We then examined the role of ZNF704 expression in cell colonies, cell cycle arrest and apoptosis and epithelial to mesenchymal transition. We additionally elucidated ZNF704 downstream signaling pathway through RNA sequencing, qRT-PCR and immunoblotting. In conclusion, ZNF704 was over-expressed in UM tissues and was inversely correlated with prognosis of UM patients by contributing not only to growth but also migration of UM cells in vitro. Mechanistically, ZNF704 function as an oncogene by down-regulates SORBS3, which we demonstrated is a tumor suppressor in UM, and in turn activates PI3K/AKT/mTOR signaling pathway.

## Supplementary Information

**Additional file 1: Table S1.** The primers used for ChIP-PCR.

**Additional file 2: Table S2.** RAS guanyl releasing protein 3 (calcium and DAG-regulated). 

**Additional file 3: Supplementary Figure 1.** The survival analysis of patients from TCGA dataset based on ZNF704 expression. The survival rate of patients in high-expression group suffered a more drastic decrease, regardless of the (A) age, (B) tumor stages, (C) Tstages, and (D) gender.

**Additional file 4: Supplementary Figure 2.** ZNF704 over-expression promotesUM cell growth. (A) qRT-PCR analysis of ZNF704 in Ctrl and ZNF704over-expressed OCM1A and C918 cells. ***p<0.001.(B) CCK8 assay was used to detect cell growth in Ctrl and ZNF704 over-expressedOCM1A and C918 cells. *p<0.05. **p<0.01. (C and D) Colony formationwas assessed in Ctrl and ZNF704 over-expressed OCM1A and C198 cells. **p<0.01. (E and F) Apoptosis wasdetected by PI/Annexin V staining in Ctrl and ZNF704 over-expressed OCM1A andC198 cells. **p<0.01.

## Data Availability

Not applicable.
