# Supplementary material for: Elevated ZNF704 expression is associated with poor prognosis of uveal melanoma and promotes cancer cell growth by regulating AKT/mTOR signaling
Source: Biomark Res. 2023 Apr 10;11:38. doi: 10.1186/s40364-023-00471-y (PMC10084591; doi:10.1186/s40364-023-00471-y)
Supplement: Supplementary file 1 — Additional file 1: Table S1. The primers used for ChIP-PCR. [file 40364_2023_471_MOESM1_ESM.pdf]

F1: CACAGTGTCTGGGAACACCA
R1: CGCGCCCCTAAAGAGGATAA

F2: CCCTTATCCTCTTTAGGGGCG
R2: AAGCACAGGGAGGAAATCAGC

F3: TCCCTTGGGCTGATTTCCTC
R3: CGGTCGTGTGCAAACAAACAC

F4: TTGCCCAGTTCACCACTCCT
R4: GGCCACTCAGAGTTTCCAGC

F5: GCGTCCCTCAAGAAGTGTCC
R5: CCAAGGCTTCCCAGGACAAA

F6: AGAAGCTTGCTGCTGGTGAG
R6: CCTTCTTATCCACGCCAGGT

SORBS3 promotor

agaagcttgctgctggtgagggggtatggccaggattcctggagtcggaagagcttcagcttcaccatcttgggagaagaagg
aagaaagtgaaagcgatgtggtccttcttagtcccaaggaccctgacaggtgggaaaaatgcgtgtctaagaaaatgggca
cctattttatccctgagggagggaagaggctacctggcgtggataagaaggtttctaagcttcaggttgggggggcagtgcact
gctgcgtccctcaagaagtgtcccagggttcccccactgctcagctgtcctccacagcaatgcccctgccatccccatctctccgcc
ttcctggagagtcagaggttcttggaaggagtcttcaaggtcatcttgttcagctccccgctcagtgcaacagttgcctttacata
gatgccccacaactggcagactcagaacccctggaatcatgtaaaaatttgtcctgggaagccttggacctgcttccctatgac
gtgtcccccagtggtcccactgctgctggtgcagctgtgtgtccacatggcagccccgcaggcgctagaggccagcaggcctatc
tgaagcttctcaacccaaacatctcctccagctttcacatgtgatgtttcaaggcaacggggagagcttctttaccagaacgag
ggagaagataagactaaagcagtaatggtggtagggactgaaaatcacttagtcttttctctactctctttctttccaaactttg
agttgagtggtgagcttggaaaacactccaaaactcctcgtggcctctggcaccagtctgacacccagtaggaatgcaggaga
ttacgttagttttcataatttagcattgatcactcccagctcgtagctaaagttgatcagtaactgttggttggttggctggtttgg
ggcttggggatccctgagccaactcaagggccagaaaggattccagagtgtttccacggtccttcaatgttccttaagcattccc
actttctgaagtgccatacagaggcttggggcagagacagggagaggggatgaccagaatggatggggagccggaagggc
cggggctctggggacccagctcagagcagcgagaacagcggaaaagcaggcagatcaaaacaaacacagtgtctgggaac
accaagaccctcctaatcccatccccgccaaagggaccccacccttatcctctttaggggcgcggcacgcccgggggggccgct
cccttgggctgatttcctccctgtgcttgcacgtgtgtgagcgtccccacgtttgcagtgtacgcgcgcgtgtttgtttgcacacgac
cggccgcgcgccgccgcaggcacaccctctcccccagcgcggaggggcggcggggccttcccctggtccgctccgccactctgcc
tgcggggcgccctcatctcgctcccggcctgtccgtctctcccggcttgcccagttcaccactcctgccccccttgtcctggctccgccc
ggttcatcgctcctgcccctgtcctggctccgctgcgaggccgctcgcttcccacactctggaaatgtcgctggaaactctgagtg
gcccgtgtcgggccgcccgacccctccccgacgccagccctgcggtaggggccgggatggcagctgggaaaccagagagggc
tccccggggaccctcggcggcccctcccggcgccgcctccccgccggccaggcctcctcggcagggggccaccagtgtccccgcg
cgcgccccgacggacggagagcactcgcacgtccctcctctccggcccagccccggcccgcagtccagatccgagacccaaact
ccgcccgccccgccgcgcgactctcacggtccggcctccggcgcgcgccccttcggcctccctcttcctgcgccggcgcccggcccg
