# Supplementary figures and images for: Elevated ZNF704 expression is associated with poor prognosis of uveal melanoma and promotes cancer cell growth by regulating AKT/mTOR signaling
Source: Biomark Res. 2023 Apr 10;11:38. doi: 10.1186/s40364-023-00471-y (PMC10084591; doi:10.1186/s40364-023-00471-y)

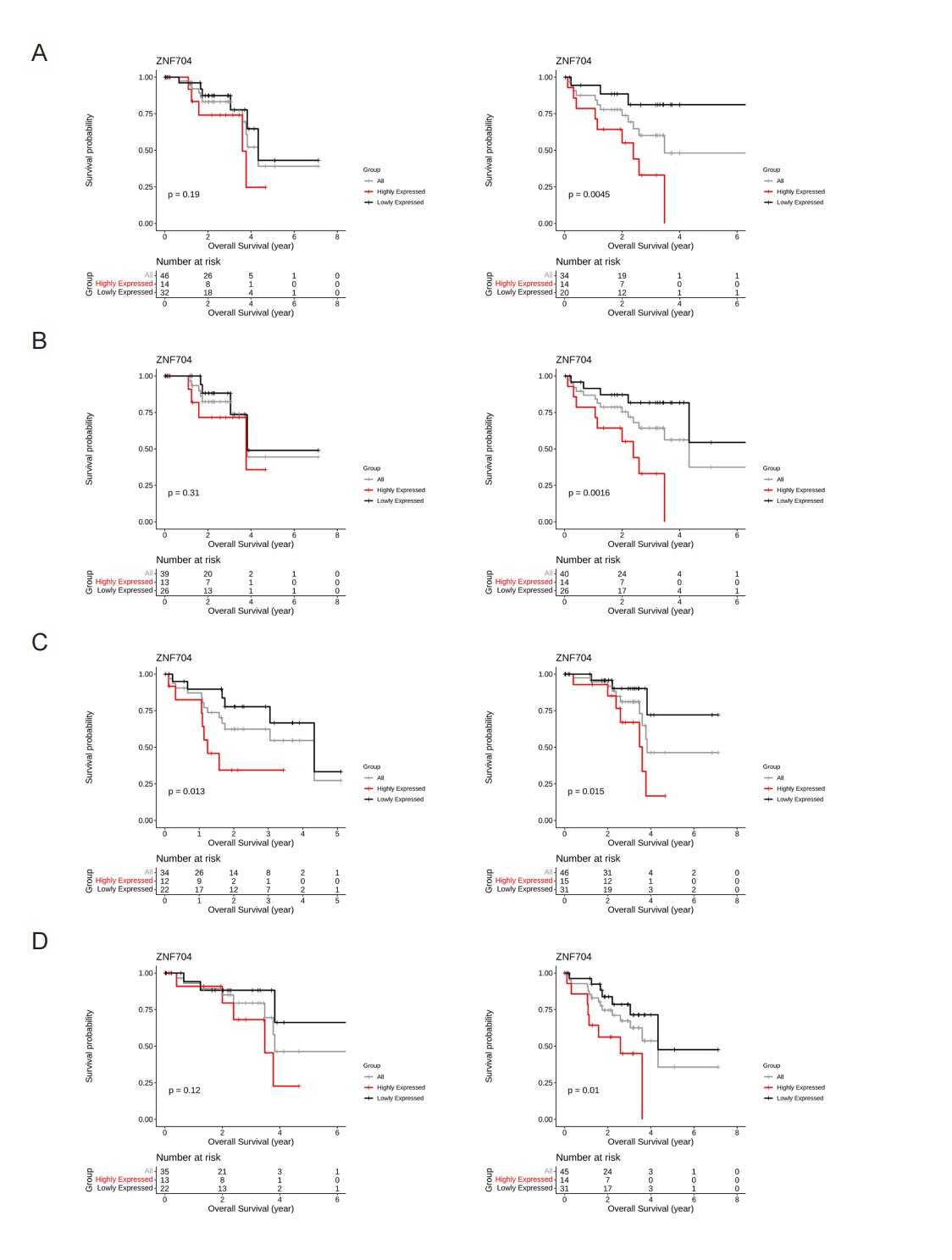

Supplement: Supplementary file 3 — Additional file 3: Supplementary Figure 1. The survival analysis of patients from TCGA dataset based on ZNF704 expression. The survival rate of patients in high-expression group suffered a more drastic decrease, regardless of the (A) age, (B) tumor stages, (C) Tstages, and (D) gender. [file 40364_2023_471_MOESM3_ESM.tif]

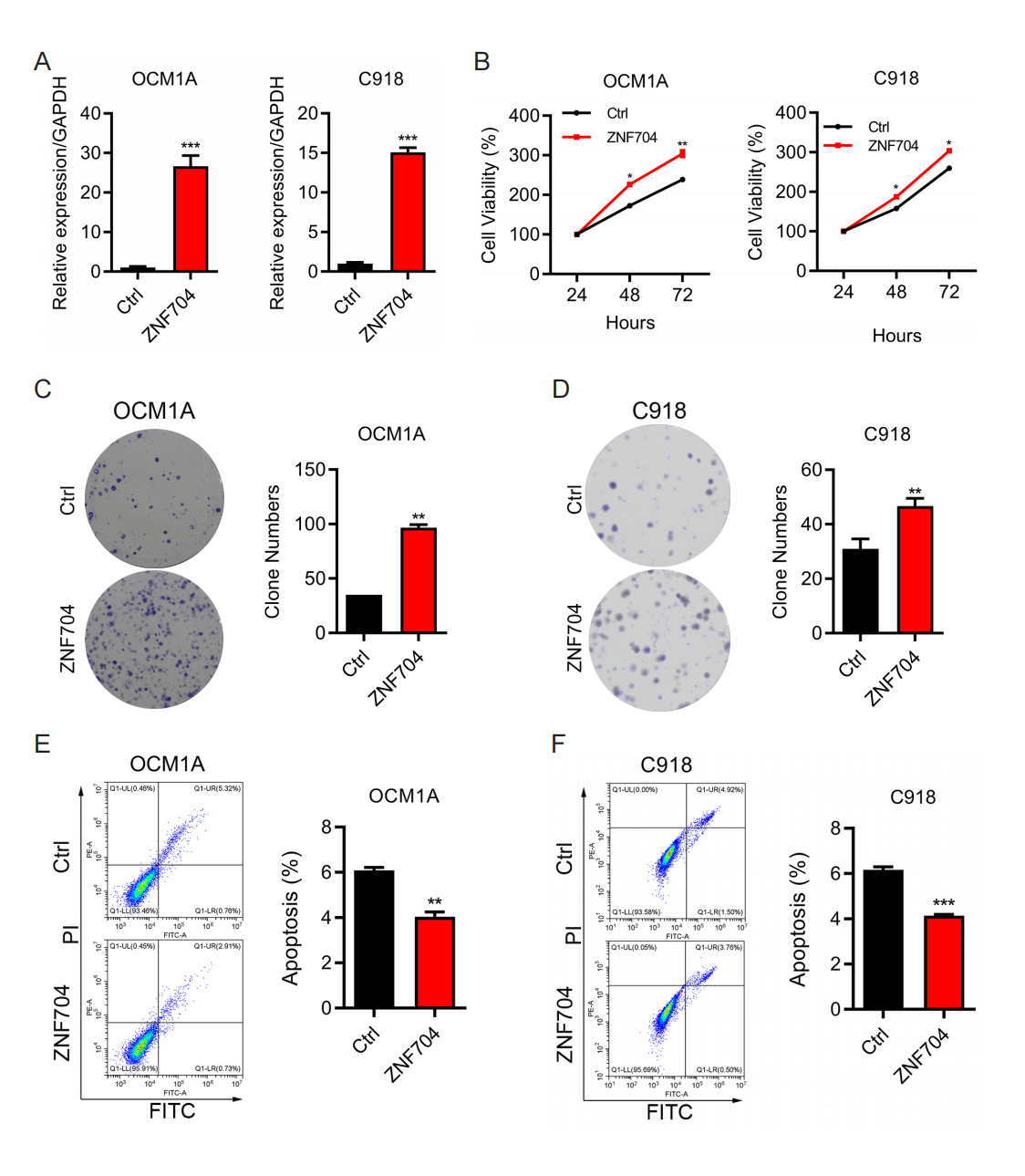

Supplement: Supplementary file 4 — Additional file 4: Supplementary Figure 2. ZNF704 over-expression promotesUM cell growth. (A) qRT-PCR analysis of ZNF704 in Ctrl and ZNF704over-expressed OCM1A and C918 cells. ***p<0.001.(B) CCK8 assay was used to detect cell growth in Ctrl and ZNF704 over-expressedOCM1A and C918 cells. *p<0.05. **p<0.01. (C and D) Colony formationwas assessed in Ctrl and ZNF704 over-expressed OCM1A and C198 cells. **p<0.01. (E and F) Apoptosis wasdetected by PI/Annexin V staining in Ctrl and ZNF704 over-expressed OCM1A andC198 cells. **p<0.01. [file 40364_2023_471_MOESM4_ESM.tif]
